# A novel measure and significance testing in data analysis of cell image segmentation

**DOI:** 10.1186/s12859-017-1527-x

**Published:** 2017-03-14

**Authors:** Jin Chu Wu, Michael Halter, Raghu N. Kacker, John T. Elliott, Anne L. Plant

**Affiliations:** National Institute of Standards and Technology, Gaithersburg, MD 20899 USA

**Keywords:** Cell image segmentation, Cell assays, Performance measure, Misclassification error rate, Total error rate, Standard error, Confidence interval, Correlation coefficient, Significance testing, Bootstrap method

## Abstract

**Background:**

Cell image segmentation (CIS) is an essential part of quantitative imaging of biological cells. Designing a performance measure and conducting significance testing are critical for evaluating and comparing the CIS algorithms for image-based cell assays in cytometry. Many measures and methods have been proposed and implemented to evaluate segmentation methods. However, computing the standard errors (SE) of the measures and their correlation coefficient is not described, and thus the statistical significance of performance differences between CIS algorithms cannot be assessed.

**Results:**

We propose the total error rate (TER), a novel performance measure for segmenting all cells in the supervised evaluation. The TER statistically aggregates all misclassification error rates (MER) by taking cell sizes as weights. The MERs are for segmenting each single cell in the population. The TER is fully supported by the pairwise comparisons of MERs using 106 manually segmented ground-truth cells with different sizes and seven CIS algorithms taken from ImageJ. Further, the SE and 95% confidence interval (CI) of TER are computed based on the SE of MER that is calculated using the bootstrap method. An algorithm for computing the correlation coefficient of TERs between two CIS algorithms is also provided. Hence, the 95% CI error bars can be used to classify CIS algorithms. The SEs of TERs and their correlation coefficient can be employed to conduct the hypothesis testing, while the CIs overlap, to determine the statistical significance of the performance differences between CIS algorithms.

**Conclusions:**

A novel measure TER of CIS is proposed. The TER’s SEs and correlation coefficient are computed. Thereafter, CIS algorithms can be evaluated and compared statistically by conducting the significance testing.

## Background

Cell image segmentation (CIS) is an essential part of quantitative imaging of biological cells, which is critical to fields such as high content screening, live cell tracking and analysis, and the analysis of subcellular structures [1–3]. Segmenting cells from fluorescent microscopy images for image-based cell assays in cytometry requires the design and development of algorithms that are optimized for a particular set of images. The performance of a CIS algorithm can affect the quantitative results derived from an image analysis pipeline.

In order to use the well-established statistical approach to evaluate and compare CIS algorithms [4] so that the statistical significance of the performance differences between CIS algorithms can be determined, besides designing a novel CIS performance measure, the standard error (SE) of the measure and the correlation coefficient of measures between two CIS algorithms must be solved. These three issues are all dealt with in this article.

**Fig. 1:**
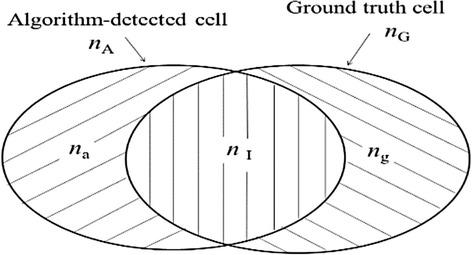
A schematic diagram showing the set-theoretic relationship between a GT cell and an AD cell where the sizes of regions are shown in terms of pixel numbers

In this study, only supervised evaluation is carried out. Cells segmented manually by experts are treated as the ground-truth (GT) cells, whereas cells segmented using an algorithm are named as the algorithm-detected (AD) cells. The set-theoretic relationship between a GT cell and its related AD cell, as shown in Fig. 1, consists of three regions: 1) the intersection region, the pixels of the GT cell identified by the algorithm; 2) the false negative (FN) region, the pixels of the GT cell missed by the algorithm; 3) the false positive (FP) region, the pixels of the AD cell that are mistakenly picked up and do not belong to the GT cell.

The numbers of pixels of the GT cell, the FN region, the AD cell, the FP region, and the intersection region are denoted by *n*_G_, *n*_g_, *n*_A_, *n*_a_, and *n*_I_, respectively, which are subject to the constraint condition *n*_G_ – *n*_g_ = *n*_A_ – *n*_a_ = *n*_I_. The FN rate is *n*_g_ / *n*_G_, and the FP rate equals *n*_a_ / *n*_A_. In this article, it is assumed that all AD cells are counted as one AD cell taken on the level of pixels if they are related to one GT cell; and all GT cells are treated as one GT cell taken on the level of pixels if they are associated with one AD cell.

Some CIS algorithms may perform better than others for cells with some specific characteristics. Many measures and methods have been proposed and implemented to evaluate the performance of segmentation methods, such as the Jaccard index, the Rand index, the Kappa statistic, and others as shown in the literature^1^ [5–9]. However, computing the SEs of the measures and their correlation coefficient is not described, although the uncertainty of the Kappa statistic was computed only for very small sizes of samples [10].

In this article, it starts with defining the misclassification error rate (MER) for segmenting a single cell in a fluorescent microscopy image. Two MERs are discussed: the average MER *r*_a_ that is an arithmetic mean of the FN rate and the FP rate, and the weighted MER *r*_w_ that is a weighted sum of these two rates using themselves as weights. The latter is more conservative than the former. Thus, the weighted MER *r*_w_ is recommended. Then, the total error rate (TER), which is a novel performance measure for segmenting all cells, is defined to be a weighted sum of all MERs, and thus statistically aggregates all MERs. The weight is the size of a GT cell divided by the total size of all GT cells in the population. Hence, the penalties on the result for an algorithm are higher if larger GT cells are not segmented correctly.

Weight is widely employed in scientific research. In our research, as stated above, error rates are used as weight in the definition of the weighted MER, and the sizes of GT cells are used as weight while defining the TER that is a consequence of using the formula of the total probability in statistics (see section “The TER for segmenting all cells”). In different applications, weight may have different concepts, for instance, in Ref. [11].

Many factors can affect how accurately a CIS algorithm detects the boundary of a cell. The cell size is one major factor. Many approaches have taken account of the size factor, but in different contexts such as the unsupervised objective evaluation methods [12].

The TER is supported by the pairwise comparisons of MERs using 106 manually segmented GT cells with different sizes and seven CIS algorithms obtained from ImageJ [13]. The CIS algorithms are IJ_Huang, IJ_RenyiEntropy, IJ_Li, IJ_MaxEntropy, IJ_Intermodes, IJ_Minimum and IJ_Triangle, numbered by 1 through 7 according to their performance levels in descending order.

**Fig. 2:**
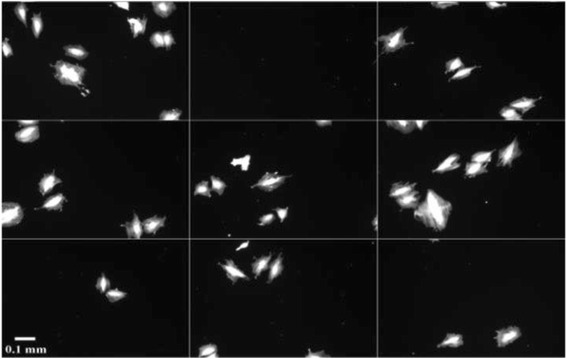
Nine fluorescent microscopy images of representative A10 rat smooth muscle cells selected from 106 manually segmented cells

The 106 cells were taken from the A10 rat smooth muscle cell line. The raw image data and manual segmentation mask data are stored at the National Institute of Standards and Technology Semantics for Biological Data Resource: Cell Image Database [14]. The imaged cells were stained TxRed c2 maleimide (Invitrogen) (5 mg/mL in DMSO stock) which labels sulfhydryl groups present on cellular proteins. Fluorescence images were acquired with an Olympus IX71 inverted microscope (Center Valley, PA) equipped with an automated stage (Ludl, Hawthorne, NY), automated filter wheels (Ludl), a Xe arc lamp fluorescence excitation source, a 10 x ApoPlan 0.4 NA objective (Olympus), and a CoolSNAP HQ CCD camera (Roper Scientific, Tucson, AZ). The filter conditions for imaging the TxRed stained cells were a 555 nm notch excitation (PN# S555_25x, Chroma Technologies, Brattleboro, VT) and a 630 nm notch emission filter (PN#S630_60m). In Fig. 2, nine fluorescent microscopy images illustrate the data used, where the cell sizes vary (concerning details of cell sizes, see section Results below).

The results derived from the TER are also consistent with the qualitative observations from the histograms of MER (see section “The TER and its SE and CI”). More importantly, the TER method is more effective than the bivariate approach using the scatter plot of the FN and FP rates, and the approach using cumulative distribution function (CDF) of MER. It is challenging to compare the performance of two CIS algorithms while the two scatter plots overlap or the two CDF curves of MER cross each other.

Then, the issue is how to estimate the SE of the TER. In this article, the SE and 95% confidence interval (CI) of the TER for CIS algorithms are computed based on the SE of MER. The calculation of the SE of MER was accomplished by using the nonparametric bootstrap method under the constraint condition shown above.

To do so, dummy scores are assigned to pixels in different regions. For a GT cell, Score 2 is assigned to all n_I_ pixels in the intersection region and Score 0 is assigned to all n_g_ pixels in the FN region. For its related AD cell, Score 0 is assigned to all n_I_ pixels in the intersection region and Score 2 is assigned to all n_a_ pixels in the FP region. And a threshold is assigned to be 1.

As a result, the score distributions of a GT cell and of its related AD cell are similar to those in the receiver operating characteristic (ROC) analysis [15–20]. Indeed, the FN rate and the FP rate with respect to the threshold 1 in the CIS are exactly the same as the cumulative probabilities of type I error and type II error in ROC analysis, respectively. And in ROC analysis, the SEs of statistics of interest can be computed using the nonparametric bootstrap method.

Our bootstrap scheme is carried out under the constraint condition *n*_G_ – *n*_g_ = *n*_A_ – *n*_a_ = *n*_I_ during bootstrap resampling, which is particular required for the CIS in the supervised evaluation involving GT cells and AD cells, as depicted in Fig. 1. In the meantime, the stochastic nature of the bootstrap method is explored in this article.

The bootstrap is applied in many areas such as evaluating stability of clusters [21]. For different applications with different statistics of interest under different circumstances, there are many different bootstrap schemes about how to resample the original data. Due to our cell sizes, nonetheless, it is computationally prohibitive to generate the exact bootstrap distribution formed by all possible bootstrap replications of the statistic of interest [10].

In this article, moreover, it demonstrates how the correlation coefficient of TERs between two CIS algorithms is computed using the synchronized resampling algorithm. Without the correlation coefficient of TERs, the two-algorithm hypothesis testing cannot be conducted [4].

All these are very useful and important in the practice of CIS. The error bars of the TER displaying the 95% CI can be used to determine whether the difference between the performance level of a CIS algorithm and a hypothesized value is statistically significant in evaluation of CIS algorithms. This is related to the one-algorithm hypothesis testing, which can simply be judged by observing whether the 95% CI of the TER contains, below, or above the hypothesized value [17].

The error bars of the TER can also be used to classify CIS algorithms into different classes in terms of performance accuracies in comparison of CIS algorithms. When the CIs overlap within the same class, because the SE of TER and the correlation coefficient can be computed, the two-algorithm hypothesis testing can be conducted to determine the statistical significance of the performance difference between two CIS algorithms. In this article, only the two-algorithm hypothesis testing will be detailed.

## Methods

### The MER for segmenting a single cell

The design of a novel performance measure in the CIS data analysis starts with defining the MER for identifying a single cell in a fluorescent image in the supervised evaluation. As stated above, the numbers of pixels, *n*_G_, *n*_g_, *n*_A_, *n*_a_, and *n*_I_, must satisfy the constraint condition,

1 $$ {n}_{\mathrm{G}} - {n}_{\mathrm{g}} = {n}_{\mathrm{A}} - {n}_{\mathrm{a}} = {n}_{\mathrm{I}}\ . $$

The FN rate *r*_fn_ and the FP rate *r*_fp_ are

2 $$ {r}_{\mathrm{fn}} = \frac{n_{\mathrm{g}}}{n_{\mathrm{G}}}\ \mathrm{and}\ {r}_{\mathrm{fp}} = \frac{n_{\mathrm{a}}}{n_{\mathrm{A}}}\ . $$

Several MERs can be defined in terms of the FN rate *r*_fn_ and the FP rate *r*_fp_. Besides “simplicity and ease of understanding” [22], conservativeness is also a criterion for defining MER in this article. Hence, two MERs are discussed as follows,

3 $$ \begin{array}{l}{r}_{\mathrm{a}} = \frac{r_{\mathrm{fn}}+{r}_{\mathrm{fp}}}{2}, \\ {}{r}_{\mathrm{w}} = \frac{r_{\mathrm{fn}}^2 + {r}_{\mathrm{fp}}^2}{r_{\mathrm{fn}}+{r}_{\mathrm{fp}}}\ .\end{array} $$

The average MER *r*_a_ is an arithmetic mean of *r*_fn_ and *r*_fp_, and the weighted MER *r*_w_ is the one using *r*_fn_ and *r*_fp_ themselves as weight so that the larger error rate pays more penalties. As *r*_fn_ and *r*_fp_ approach to zero, *r*_w_ goes to zero as well. Both *r*_a_ and *r*_w_ vary in the region [0, 1]: 0 stands for the best segmentation when an AD cell is identical to the related GT cell, and 1 means the worst classification when an AD cell and the associated GT cell are disjoint.

First, it is trivial to prove that the arithmetic mean of the FN rate *r*_fn_ and the FP rate *r*_fp_ is greater than or equal to the geometric mean $\sqrt{r_{\mathrm{fn}}{r}_{\mathrm{fp}}}$, which is subsequently greater than or equal to the harmonic mean 2*r*_fn_ *r*_fp_ / (*r*_fn_ + *r*_fp_) [23]. These three means are all equal if and only if *r*_fn_ = *r*_fp_. So, the arithmetic mean leads to more conservative (i.e. larger) estimates for the error rates.

Further, when either FN rate r_fn_ or FP rate r_fp_ approaches zero, both geometric and harmonic means go to zero, which indicates perfect segmentation in the supervised evaluation, no matter how much the other error rate is. Under such circumstances, however, the arithmetic mean approaches half of the other error rate.

**Fig. 3:**
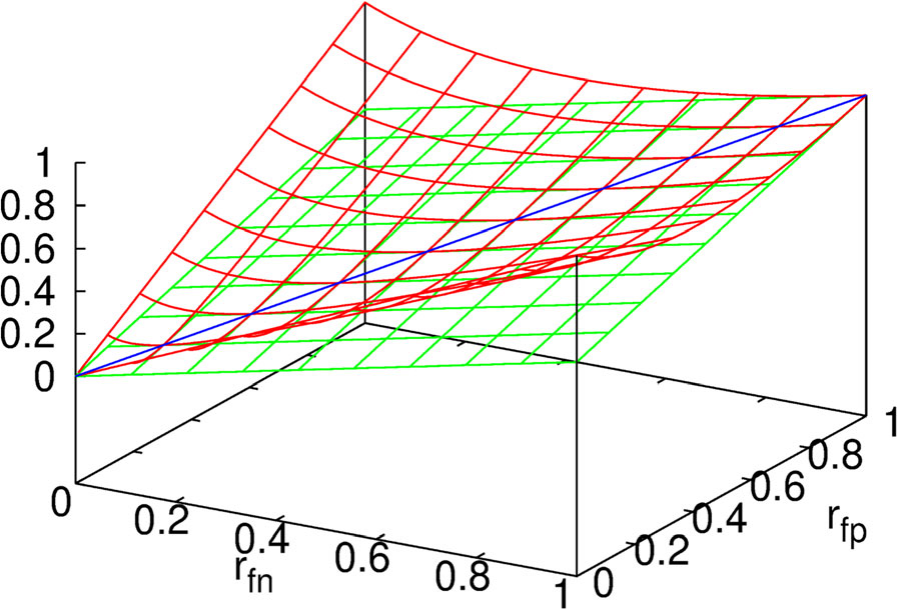
The average MER *r*_a_ is a plane (*green*) and the weighted MER *r*_w_ is a surface (*red*) with respect to the FN rate *r*_fn_ and the FP rate *r*_fp_. They are tangent along a straight line (*blue*)

Second, the weighted MER *r*_w_ is compared with the average MER *r*_a_. Both of them are simple and easy to understand. However, as stated above, the weighted MER *r*_w_ does penalize errors because of using the error rate as weight [22]. Moreover, it is trivial to prove from Eq. (3) that *r*_w_ = *r*_a_ if and only if *r*_fn_ = *r*_fp_; otherwise, *r*_w_ > *r*_a_. This can also be seen in Fig. 3, where *r*_a_ is a plane in green and *r*_w_ is a surface in red as functions of *r*_fn_ and *r*_fp_. The red surface is above the green plane except they are tangent along a straight line in blue. In other words, the weighted MER *r*_w_ is a more conservative measure than the average MER *r*_a_.

If an algorithm segments a small GT cell completely with a relatively very large AD cell, then *r*_fn_ = 0 and *r*_fp_ → 1. If an algorithm detects a large GT cell with a relatively very small AD cell located completely inside the GT cell, then *r*_fp_ = 0 and *r*_fn_ → 1. They all imply that *r*_w_ → 1 but *r*_a_ → 1/2 due to Eq. (3). These two cases can also be seen from Fig. 3. Indeed, under these two circumstances, the MER should be much larger than 1/2 and close to 1. It indicates that the weighted MER *r*_w_ can deal with these special cases better than the average MER *r*_a_, although in reality such special cases occur quite rarely.

Both *r*_w_ and *r*_a_ can be expressed as functions of the size of the intersection region *n*_I_ so that a simulation can be carried out. The former is a linear function with negative slope. The latter is a more complicated function that decreases first and then increases as *n*_I_ increases if *n*_G_ ≠ *n*_A_; but is the same function as the former if *n*_G_ = *n*_A_. Both functions are symmetric with respect to *n*_G_ and *n*_A_. All these imply that *r*_w_ and *r*_a_ behave differently when *n*_I_ varies. That is, when an AD cell approaches to the related GT cell, *r*_w_ decreases first and then increases if *n*_G_ ≠ *n*_A_, but *r*_a_ always decreases. One may ask: Why cannot a CIS algorithm segment a GT cell completely when the AD cell is getting so close to it?

Based on these analyses, the weighted MER *r*_w_ rather than the average MER *r*_a_ is recommended. Thus, in this article, only those results computed using the weighted MER *r*_w_ will be shown. Nonetheless, as far as computational results are concerned, qualitatively speaking, there is not too much discrepancy between the two MERs. These will be mentioned in the following text.

**Fig. 4:**
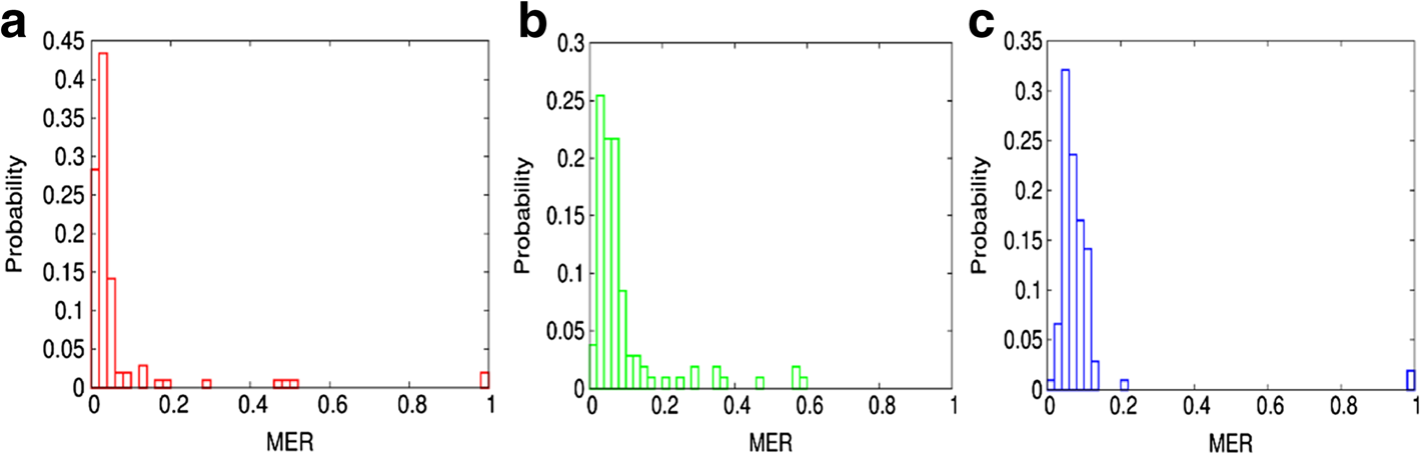
Histograms of the weighted MERs generated using Algorithms 1 (**a**), 2 (**b**), and 3 (**c**)

Figure 4 shows the histograms of the weighted MERs generated using Algorithms 1, 2, and 3 to segment 106 cells (see section Results). These three histograms overlap each other. However, they shift towards larger MER from Algorithm 1 to 3, suggesting that Algorithm 1 may be better than Algorithm 2 that in turn may be better than Algorithm 3. If the average MERs are employed, the relationship of the three histograms remains the same.

### The TER for segmenting all cells

As pointed out in section Background, in this article, it is assumed that all AD cells are counted as one AD cell taken on the level of pixels if they are related to one GT cell; and all GT cells are treated as one GT cell taken on the level of pixels if they are associated with one AD cell. Hence, generally speaking, segmenting a cell in fluorescent microscopy images is an exclusive event with respect to detecting other cells. Then, to measure the performance level of a CIS algorithm, based on the formula of the total probability in statistics [4, 24, 25], the TER *ε* is defined to be a weighted sum of all MERs,

4 $$ \begin{array}{l}\varepsilon \equiv \Pr (CIS) = {\displaystyle \sum_{i=1}^N} \Pr \left( CIS\ \Big|{C}_i\right)\  \Pr \left({C}_i\right)\ \\ {}\kern5.25em  = {\displaystyle \sum_{i=1}^N} ME{R}_i \times \frac{S_i}{{\displaystyle {\sum}_{j=1}^N}{S}_j}, \end{array} $$

where *N* is the total number of GT cells, Pr(*CIS*) stands for the total probability of making misclassification errors while using an algorithm to detect all cells in a fluorescent image, the conditional probability Pr(*CIS* | *C*_i_) means the MER while segmenting the i-th GT cell in the image which is denoted by MER_i_, and Pr(*C*_i_) is the probability of the occurrence of the i-th GT cell that is assumed to be the ratio of the size of the i-th GT cell *S*_i_ to the total sizes of all GT cells. Hence, the TER ε statistically aggregates all cells’ MERs,

It can be proven that the TER *ε* varies in the region [0, 1], where 0 stands for the best performance of the algorithm and 1 means the worst performance. As shown in Eq. (4), the cell sizes are used as weights. So, it can ensure that it penalizes errors and the penalties for misclassifying cells are proportional to the sizes of cells [22].

### The SE and 95% CI of TER

First, the SE of MER is computed using a bootstrap method. Second, based on that, the SE and 95% CI of TER are calculated. Third, the variation of the SE of TER is explored due to the stochastic nature of the bootstrap approach.

#### The SE of MER for segmenting a single cell

The MER for segmenting a single GT cell consists of the FN rate and the FP rate, and these two rates are formed by the numbers of pixels in different regions as shown from Eq. (1) to Eq. (3). Based on the assignment of dummy Scores 0 and 2 described in section Background, the score set for a GT cell is expressed as,

5 $$ \mathbf{G} = \left\{{\mathrm{g}}_{\mathrm{i}}=0\left|\ \mathrm{i}=1, \dots, {n}_g;{\mathrm{g}}_{\mathrm{i}} = 2\ \right|\mathrm{i}={n}_{\mathrm{g}}+1, \dots, {n}_{\mathrm{G}}\right\}, $$

and the score set for its related AD cell is denoted as,

6 $$ \mathbf{A} = \left\{{\mathrm{a}}_{\mathrm{i}}=2\ \left|\ \mathrm{i}=1, \dots, {n}_{\mathrm{a}};\ {\mathrm{a}}_{\mathrm{i}}=0\ \right|\mathrm{i}={n}_{\mathrm{a}} + 1, \dots, {n}_{\mathrm{A}}\right\}, $$

where the constraint condition Eq. (1) must hold true.

There are five possibilities regarding the set-theoretic relationship between a GT cell and its associated AD cell: 1. the two cells are disjoint, 2. they are completely overlapped, 3. the GT cell completely contains the AD cell, 4. the AD cell completely contains the GT cell, 5. they are partially overlapped. Case 5 occurs most often in this study.

In the following, the bootstrap algorithm for computing the SE of MER is presented in a way to deal with Cases 4 and 5, in which both *n*_a_ and *n*_I_ = *n*_A_ – *n*_a_ are positive. Thus, the bootstrap random resampling with replacement (WR) can be legitimately applied to the score set of an AD cell in Eq. (6) [15–20]. Here is the nonparametric one-sample bootstrap algorithm of computing the SÊ of MER for segmenting a single cell.


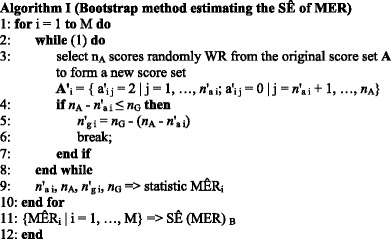


where M is the number of bootstrap replications. As shown from Step 1 to 10, this algorithm runs M times. In the i-th iteration, there is an endless **while** loop from Step 2 to 8. In this loop, *n*_A_ scores are randomly selected WR from the original score set **A** in Eq. (6) to form a new score set **A'**_i_, which contains *n*'_a i_ Score 2 forming a new FP region and *n*_A_ - *n*'_a i_ Score 0 forming a new intersection region, as shown in Step 3. Then, the constraint condition Eq. (1) needs to be checked. If the size of the new intersection region is less than or equal to the size of the GT cell in Step 4, the size of the new FN region, *n*'_g i_, is determined in Step 5 and the **while** loop breaks in Step 6; otherwise, the endless **while** loop continues.

After the **while** loop breaks, the i-th estimated MÊR_i_ can be obtained in Step 9 from the new sizes of FP region and FN region, *n*'_a i_ and *n*'_g i_, and the original sizes of the AC cell and the GT cell, *n*_A_ and *n*_G_, using Eqs. (2) and (3). Finally, after M iterations, in Step 11, a bootstrap distribution is formed by the bootstrap replications of the MER, i.e., {MÊR_i_ | i = 1, …, M}, and then the standard error SÊ(MER)_B_ can be estimated using the sample standard deviation of this distribution.

Algorithm I can be easily converted to handling Case 3 in which there is no pixel in the FP region, if the score set of the GT cell in Eq. (5) is resampled. So, the scores and sets related to an AD cell should be replaced by the scores and sets related to a GT cell in Step 3, and the statements in Steps 4 and 5 should be changed to “**if** *n*_G_ - *n*'_g i_ ≤ *n*_A_ **then**” and “*n*'_a i_ = *n*_A_ - (*n*_G_ - *n*'_g i_)” accordingly.

In Case 1, both *r*_fn_ and *r*_fp_ equal 1 and thus both *r*_a_ and *r*_w_ are 1. In Case 2, both *r*_fn_ and *r*_fp_ equal 0 and thus both *r*_a_ and *r*_w_ are 0. In these two cases, the estimates of SEs of both *r*_a_ and *r*_w_ are assumed to be 0, meaning that there is no variation associated with such MERs. So, the output of Algorithm I for Cases 1 and 2 is assumed to be zero.

The remaining issue is to determine how many iterations this bootstrap algorithm needs to run in order to reduce the bootstrap variance and ensure the accuracy of the computation. The appropriate number M of the bootstrap replications was determined to be 2000 based on our empirical bootstrap variability studies in ROC analysis [17–20].

#### The SE and 95% CI of TER for segmenting all cells

After the bootstrap estimated SÊ of MER for segmenting each GT cell is computed, assuming that detecting and segmenting different GT cells in fluorescent microscopy images are mutually independent, the estimated variance of the TER *ε* for detecting all GT cells can be obtained based on Eq. (4),

7 $$ \mathrm{V}\mathrm{a}\mathrm{r}\ \left(\varepsilon \right) = {\displaystyle \sum_{i=1}^N}{\left(\frac{S_i}{{\displaystyle {\sum}_{j=1}^N}{S}_j}\right)}^2 \times \mathrm{S}\widehat{\mathrm{E}}{\left(\mathrm{MER}\right)}_{\mathrm{B}\  i}^2 $$

where *N* is the total number of cells, *S*_i_ is the size of the i-th GT cell, and SÊ(MER)_B i_ stands for the bootstrap estimated SÊ of MER for segmenting the i-th GT cell.

Then, the estimated SÊ of the TER *ε* is defined to be the square root of Var (*ε*). Again from Eq. (4), generally speaking, if no independent random variable dominates the others, the distribution of the TER can be assumed to be approximately normal because of the central limit theorem [26]. Thereafter, the estimated 95% CÎ of the TER *ε* can be obtained by adding and subtracting 1.96 times the estimated SÊ.

#### The variation of the SE of TER

The nature of the bootstrap method is stochastic. Each execution of the bootstrap algorithm may result in different SÊs of MERs and thus different SÊs of a TER. It is necessary to investigate how much the estimated SÊ of the TER varies. Hence, a distribution of such estimates needs to be generated. Here is the algorithm to create such a distribution.


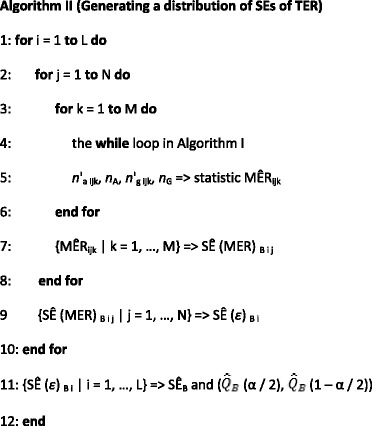


where M is the number of bootstrap replications, N is the total number of cells, L is the number of the Monte Carlo iterations, and Step 4 is the **while** loop in Algorithm I from Step 2 to 8.

From Step 3 to 7, Algorithm I is employed to compute the SÊ (MER)_B_ of an MER for segmenting a single GT cell. From Step 2 to 8, Algorithm I is used to compute SÊs of MERs for all N GT cells. Thus, at Step 9, an estimated SÊ (*ε*)_B_ of the TER *ε* for detecting all GT cells is calculated using Eq. (7).

Such a process is executed in L times from Step 1 to 10. After L iterations, at Step 11, L estimated SÊ (*ε*)_B i_ of the TER *ε* are generated and constitute a distribution. Thereafter, the estimated SÊ_B_ and the (1–α)100% CÎ (${\widehat{Q}}_B$(α/2), ${\widehat{Q}}_B$(1–α/2)) at the significance level α of the distribution can be computed.

The estimated α/2 100% and (1–α/2) 100% quantiles of the distribution are calculated using the Definition 2 of quantile in Ref. [27]. That is, the sample quantile is obtained by inverting the empirical distribution function with averaging at discontinuities. If 95% CÎ is of interest, then α is set to be 0.05.

Finally, the number of the Monte Carlo iterations L needs to be determined in order to guarantee the accuracy of the Monte Carlo computation. Based on our previous studies, to create a stable distribution, it is enough that the repeated process described above be executed 500 times, i.e., L = 500 [17–20].

### Significance testing

The two-algorithm hypothesis testing is carried out by the Z test, since the TER can be assumed to be normally distributed as pointed out above [4].

#### Two-algorithm hypothesis testing

Let T_A_ and T_B_ denote the TERs for CIS Algorithms A and B, respectively. Then, the null and alternative hypotheses are

8 $$ \begin{array}{l}{H}_{\mathrm{o}}:{T}_{\mathrm{A}}={T}_{\mathrm{B}}\\ {}{H}_{\mathrm{a}}:{T}_{\mathrm{A}}\ne {T}_{\mathrm{B}}\end{array} $$

Based on the normality assumption, the general Z statistic for two-algorithm hypothesis testing is

9 $$ Z=\frac{\widehat{T}\mathrm{A}\ \hbox{-}\ \widehat{T}\mathrm{B}}{\sqrt{{\mathrm{SE}}^2\left(\widehat{T}\mathrm{A}\right) + {\mathrm{SE}}^2\left(\widehat{T}\mathrm{B}\right)\ \hbox{-}\ 2\ \rho\ \mathrm{SE}\left(\widehat{T}\mathrm{A}\right)\ \mathrm{SE}\left(\widehat{T}\mathrm{B}\right)}} $$

where $\widehat{T}\mathrm{A}$ and $\widehat{T}\mathrm{B}$ are two estimated TERs, SE($\widehat{T}\mathrm{A}$) and SE($\widehat{T}\mathrm{B}$) stand for their SEs, respectively, and ρ is the correlation coefficient between $\widehat{T}\mathrm{A}$ and $\widehat{T}\mathrm{B}$.

#### An algorithm for computing the correlation coefficient

This algorithm is based on the synchronized resampling approach. The two algorithms segment the same set of GT cells. The MERs of two CIS algorithms due to segmenting cells with the same ordinal number in the set of GT cells co-vary. As a result, the two TERs of any two CIS algorithms are correlated. The tendency of obtaining higher or lower MERs for segmenting the same GT cell could be different. Therefore, the correlation between the TERs of any two CIS algorithms may be positive or negative.

Using the notations in Eqs. (5) and (6), a score set that an Algorithm A segments the i-th GT cell with size *n*_G i_ in the set of *N* GT cells and generates *n* ^A^_g i_, *n* ^A^_A i_, and *n* ^A^_a i_ accordingly is denoted by

10 $$ {\mathbf{C}}^A = \left\{{n}_{\mathrm{G}\ \mathrm{i}},{n^{\mathrm{A}}}_{\mathrm{g}\ \mathrm{i}},{n^{\mathrm{A}}}_{\mathrm{A}\ \mathrm{i}},{n^{\mathrm{A}}}_{\mathrm{a}\ \mathrm{i}}\Big|\mathrm{i}=1, \dots, N\right\}, $$

from which a TER can be computed using Eqs. (2) through (4). All CIS algorithms segment the same set of *N* GT cells. Thus, the size of the i-th GT cell, i.e., n_G i_, is the same for all CIS algorithms. This correlates TERs of different algorithms.

An algorithm for computing the correlation coefficient of the TERs for CIS Algorithms A and B is as follows.


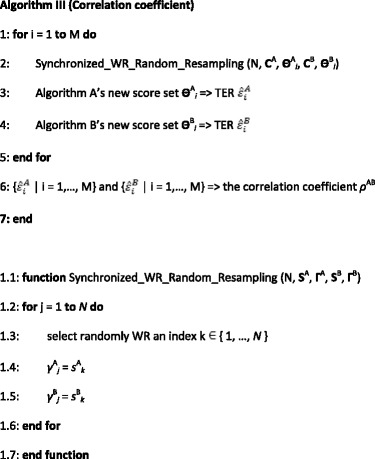


where *s* ^A^_*k*_, *γ* ^A^_*j*_, *s* ^B^_*k*_, and *γ* ^B^_*j*_ are members of the score sets **S** ^A^, **Γ** ^A^, **S** ^B^, and **Γ** ^B^, respectively. Based on our bootstrap variability studies, the number of iterations M is set to be 2000 [17–20].

From Step 1 to 5, this algorithm runs M iterations. In Step 2 of the i-th iteration, the synchronized WR random resampling is carried out on the two score sets **C** ^A^ and **C** ^B^ of Algorithms A and B to generate two new score sets **Θ** ^A^_*i*_ and **Θ** ^B^_*i*_.

From Step 1.1 to 1.7, during the resampling iterations, if a member with index k in **S** ^**A**^ is WR randomly selected, then the member with the same index k in **S** ^**B**^ is also selected. That is, a GT cell with the same ordinal number k in the set of *N* GT cells is selected. Thus, such synchronized selections guarantee that all co-varying members in score sets between the two CIS algorithms are selected simultaneously. Hence, the correlation of the TERs between the two algorithms is preserved.

After resampling, in Step 3 (4), the *i*-th estimated TER ${\widehat{\varepsilon}}_i^A$ (${\widehat{\varepsilon}}_i^B$) of Algorithm A (B) is computed from the new score set **Θ** ^A^_*i*_ (**Θ** ^B^_*i*_). Finally in Step 6 after M iterations, the correlation coefficient *ρ* ^AB^ of the TERs of Algorithms A and B is computed from the two sets of correlated TERs.

A synchronized random resampling is involved here. Thus, this algorithm needs to run multiple times to reduce the computational fluctuation, if the *p*-value is not considerably different from the critical values, such as 5%, 1%, etc. To be more conservative, in this article, the average out of 10 runs was taken to be the resultant correlation coefficient for significance testing.

## Results

**Fig. 5:**
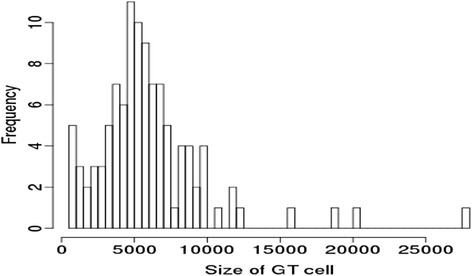
The histogram of the sizes of all 106 GT cells

The dataset consisted of 106 cells with different sizes, which were manually segmented as GT cells. Figure 5 shows the histogram of the cell size. The sizes ranged from 647 up to 27,562 pixels with the mean size at 6062 pixels. The variation of cell sizes was quite large. Thus, the cell sizes must be taken into account while evaluating CIS algorithms.

The seven CIS algorithms in ImageJ were implemented. As stated above, the estimated SÊs of MERs in Cases 1 and 2 are zero, which can lower the estimate of the SE of TER for detecting all GT cells. Only Algorithm 4 created one Case 1 and Algorithm 7 produced three; and no algorithm generated Case 2.

### Pairwise comparisons to support the TER

Two CIS algorithms generate two weighted MERs while segmenting the same GT cell, and these two weighted MERs are compared. Table 1 shows the relationship in terms of the numbers of “less than” (<), “greater than” (>), and “equal to” (=) between such two weighted MERs while using two algorithms to segment all 106 GT cells. For instance, comparing Algorithms 1 with 2, for 87 GT cells, the weighted MERs generated using Algorithm 1 are less than those created using Algorithm 2; and for only 19 GT cells, the “greater than” occurs otherwise. This indicates that the performance of Algorithm 1 is better than the performance of Algorithm 2.

**Table 1:** Comparisons of the weighted MERs generated using two algorithms for all 106 cells in terms of the numbers of inequalities and equalities

Algorithm		the number of		
		<	>	=
1	2	87	19	0
2	3	57	49	0
3	4	68	38	0
4	5	59	47	0
5	6	101	5	0
6	7	79	27	0

Further, in Table 1, the relationship of “better than” is transitive. For example, the performance of Algorithm 1 is also better than the performance of Algorithm 3, and so on. Indeed, while comparing Algorithms 1 with 3, there are 91 “<” and 15 “>”. As a result, the test of pairwise comparisons of MERs in this article was conducted between any two out of seven CIS algorithms. Table 1 shows that the performance is degraded in the ascending order of the CIS algorithms.

If the average MERs are employed, qualitatively speaking, the relationship among these CIS algorithms in terms of numbers of “<”, “>”, and “=” stays the same.

### The TER and its SE and CI

Table 2 shows the estimated TÊRs, SÊs (relative errors) and 95% CÎs of TERs for the seven CIS algorithms, when the weighted MERs are employed. The smaller the estimated TÊR ε is, the better the performance is. The order of the algorithms in Table 2 is consistent with the one in Table 1. It indicates that the TER constructed on all MERs and using the cell sizes as weights is fully supported by the results derived directly from the pairwise-comparison test of MERs using 106 GT cells with different sizes and seven CIS algorithms taken from ImageJ.

**Table 2:** The estimated TÊRs, SÊs (relative errors) and 95% CÎs of TERs for the seven CIS algorithms, in which the weighted MERs are employed

Alg.	TÊR	SÊ (relative error)	95% CÎ of TER
1	0.057524	0.000893 (3.04%)	(0.055775, 0.059274)
2	0.066889	0.000093 (0.27%)	(0.066707, 0.067071)
3	0.089363	0.000674 (1.48%)	(0.088042, 0.090684)
4	0.105096	0.000061 (0.11%)	(0.104976, 0.105215)
5	0.171153	0.001721 (1.97%)	(0.167780, 0.174526)
6	0.173513	0.000868 (0.98%)	(0.171812, 0.175213)
7	0.224444	0.000095 (0.08%)	(0.224257, 0.224631)

Moreover, regarding Algorithms 1, 2, and 3, their estimated TÊR ε shown in Table 2 are qualitatively consistent with the observations in Fig. 4, where the histograms of the weighted MERs for these three algorithms shift gradually towards larger MER.

**Fig. 6:**
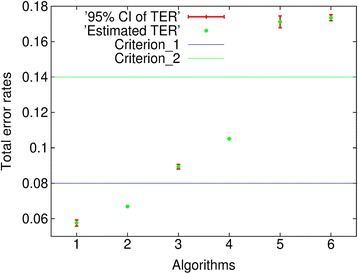
The error bars of TER displaying 95% CIs along with estimated TERs for six CIS algorithms, in which the weighted MERs are employed, with two criteria set at 0.08 and 0.14 that statistically classify CIS algorithms

The relative error of the TER can be defined as “1.96 × SÊ / TÊR”, where 1.96 is the Z score corresponding to 95% CI. Thus, the ranges of relative errors are between 0.08% and 3.04%. Most importantly, Fig. 6 shows the error bars of the TER displaying the 95% CÎs along with estimated TÊRs for six CIS algorithms, when the weighted MERs are employed. Algorithm 7 is not included due to large TÊR.

If the average MERs are employed, the corresponding estimated TÊRs are smaller, which is consistent with what was discussed in sections “The MER for segmenting a single cell” and “The TER for segmenting all cells” (see section Discussion). But qualitatively speaking, except that the 95% CÎ of TER of Algorithm 1 contains the one of Algorithm 2, everything else stated here remains the same.

### The variation of the estimated SÊ of the TER

The nature of the bootstrap method is stochastic. Algorithm II was used to deal with this issue. Table 3 shows the means, SÊs (relative errors), and 95% CÎs of the estimated SÊs of TÊRs for the seven CIS algorithms, when the weighted MERs are employed. The relative error is defined as “1.96 × SE / mean” to take account of all estimates occurring in the estimated 95% CÎ. All 95% CÎs are quite narrow. The largest relative error is 1.87%.

**Table 3:** The means, SÊs (relative errors), and 95% CÎs of the estimated SÊs of TERs for the seven CIS algorithms, in which the weighted MERs are employed

Alg.	Mean	SÊ (relative error)	95% CÎ of SÊ of TER
1	0.000903	0.000007 (1.47%)	(0.000890, 0.000916)
2	0.000093	0.000000 (0.57%)	(0.000092, 0.000093)
3	0.000668	0.000006 (1.87%)	(0.000657, 0.000682)
4	0.000061	0.000000 (0.99%)	(0.000060, 0.000061)
5	0.001712	0.000012 (1.36%)	(0.001689, 0.001735)
6	0.000874	0.000006 (1.36%)	(0.000863, 0.000886)
7	0.000096	0.000000 (0.97%)	(0.000095, 0.000097)

**Fig. 7:**
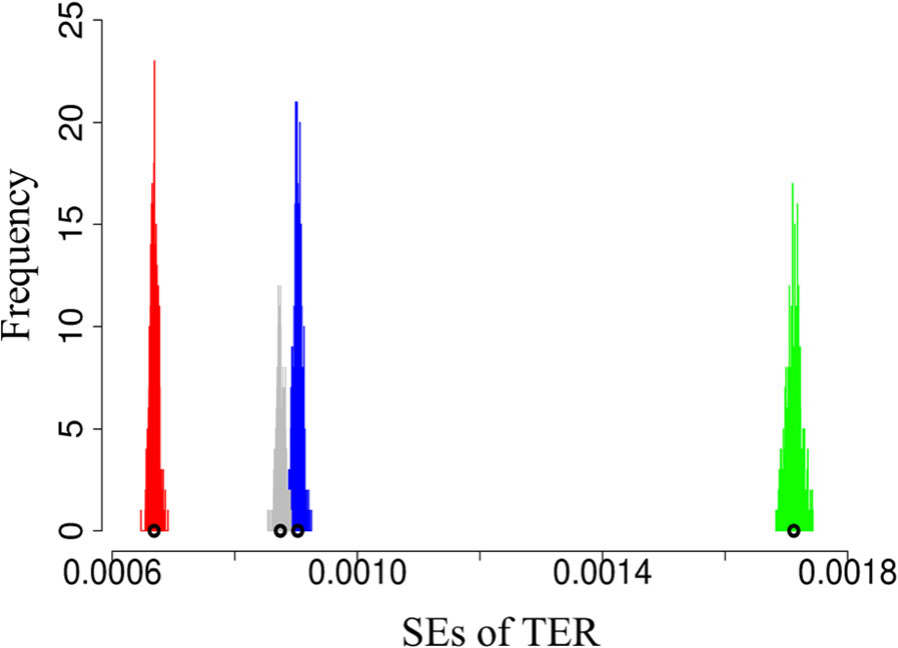
The histograms of the estimated SÊs of TÊRs for four CIS Algorithms 1 (blue), 3 (red), 5 (green), and 6 (gray), in which the weighted MERs are employed. The black circle stands for the estimated mean of the distribution

Figure 7 shows the histograms of the estimated SÊs of TERs for CIS Algorithms 1 (blue), 3 (red), 5 (green), and 6 (gray), when the weighted MERs are employed. The histograms of other three algorithms are too narrow to draw. The widths of all distributions are very narrow, demonstrating that the results are quite stable.

Taking Algorithm 1 as an example whose TÊR in Table 2 is the smallest and relative error is the largest, and using Algorithm 1’s estimated 95% CÎ of SÊs of TERs in Table 3, it can be calculated that the relative errors of TÊR may vary between 3.03% and 3.12%.

It is worth mentioning that in Table 2, all estimated SÊs of TERs were calculated by a random execution of the stochastic bootstrap method while computing the SÊs of MERs. However, they all correspondingly fall in the 95% CÎ of the estimated SÊs of TERs shown in Table 3.

Again, if the average MERs are employed, qualitatively speaking, nothing stated here is changed. For instance, the relative errors of TÊR for Algorithm 1 varies between 5.31% and 5.56%, which is also very narrow.

### Significance testing

CIS algorithms may be statistically classified into different classes in terms of performance accuracies using the error bars. This provides a basis for identifying algorithms that are quantitatively similar to one another. For instance, as shown in Fig. 6, if the criteria of performance accuracies are set to be at 0.08 and 0.14, respectively, then Algorithms 1 and 2 are classified to be in the first class, Algorithms 3 and 4 are in the second class, and Algorithms 5 and 6 are in the third class. This is because their error bars, i.e., the 95% CÎs of TER, do not cross the criteria. Otherwise, the one-algorithm hypothesis testing needs to be taken. Certainly, the criteria are set depending on the circumstances being dealt with.

When two error bars do not overlap, for example, for Algorithms 1 and 2 as depicted in Fig. 6, the performance level of the CIS algorithm corresponding to the lower error bar is better than the other one. When two error bars overlap, for example, for Algorithms 5 and 6, the two-algorithm hypothesis testing is necessary to determine the statistical significance of performance difference.

To demonstrate, the hypothesis testing is conducted on Algorithms 1 and 2, as well as on Algorithms 5 and 6. The corresponding correlation coefficients of TERs computed using Algorithm III are 0.215203, and 0.370554, respectively. Then, using the TERs and their SEs shown in Table 2, the Z-test two-tailed p-values are 0%, and 14.4% accordingly.

Using 5% as a critical p-value, these p-values show that the performance level of Algorithm 1 is better than the performance level of Algorithm 2, even though they are in the same first class. However, the difference in terms of performance accuracy between Algorithms 5 and 6 is not statistically significant.

If the average MERs are used, it is pointed out above that the 95% CÎ of TER of Algorithm 1 contains the one of Algorithm 2. This is consistent with the result of conducting the hypothesis testing. The p-value is 11.4%, which shows that the performance difference between Algorithms 1 and 2 is not statistically significant. This is the only difference qualitatively speaking between using the weighted MERs and the average MERs on our CIS datasets. Nonetheless, as analyzed in section “The MER for segmenting a single cell”, the weighted MER is recommended.

## Discussion

The MER for segmenting a single cell in the supervised evaluation can be defined in several ways. It is hard to find one without any disadvantages [22]. Simplicity, ease of understanding, penalizing errors, conservativeness, and dealing with special cases are the criteria of choosing MER in this article. Based on our analyses, the weighted MER *r*_w_ is recommended. Certainly, those special cases, in which either *r*_fn_ = 0 and *r*_fp_ → 1 or *r*_fp_ = 0 and *r*_fn_ → 1 presented in section “The MER for segmenting a single cell”, may occur quite rarely in practice.

For the seven CIS algorithms employed in section “The TER and its SE and CI”, if the average MER *r*_a_ is used, the estimated TÊRs are 0.035842, 0.037330, 0.046528, 0.058023, 0.086210, 0.087080, and 0.127707. As expected, they are correspondingly smaller than those if the weighted MER *r*_w_ is employed (see section “The TER and its SE and CI”).

For the sake of discussion, if the MER is defined to be “the proportion of objects which it misclassifies” [22], *r*_3_ = (*n*_g_ + *n*_a_) / (*n*_G_ + *n*_A_) = (*n*_G_ x *r*_fn_ + *n*_A_ x *r*_fp_) / (*n*_G_ + *n*_A_), which is a function of three independent variables under the constraint condition Eq. (1). Here are three observations. If *n*_G_ = 4694, *n*_A_ = 5276, *n*_g_ = 16, and *n*_a_ = 598, then *r*_w_ = 0.110134, *r*_a_ = 0.058376, and *r*_3_ = 0.061585, where *r*_3_ is very close to *r*_a_ but almost half of *r*_w_. If *n*_G_ = 1420, *n*_A_ = 3492, *n*_g_ = 5, and *n*_a_ = 2077, then *r*_w_ = 0.591308, *r*_a_ = 0.299155, and *r*_3_ = 0.423860, where *r*_3_ is in the middle of *r*_w_ and *r*_a_. If *n*_G_ = 6155, *n*_A_ = 14, *n*_g_ = 6141, and *n*_a_ = 0 (i.e., one of the above special cases), then *r*_w_ = 0.997725, *r*_a_ = 0.498863, and *r*_3_ = 0.995461, where *r*_3_ is close to *r*_w_ but about twice as large as *r*_a_. Hence, the MER *r*_3_ is not discussed further in this article.

The SE of the average MER *r*_a_ may be estimated analytically, because the correlation coefficient between the FN rate *r*_fn_ and the FP rate *r*_fp_ in the CIS application is 1 as proven in the following.

For a GT cell and its related AD cell, assuming they are not disjoint, once the size of the FN region increases or decreases by one pixel, the size of the FP region will increase or decrease by one pixel accordingly due to the constraint condition Eq. (1). Using the notations in section “The MER for segmenting a single cell”, the correlated pairs of the FN rate r_fn_ and the FP rate r_fp_ are

11 $$ \left(\ {r}_{\mathrm{fn}\  i},\ {r}_{\mathrm{fp}\  i}\right) = \left(\frac{n_{\mathrm{g}}+ i}{n_{\mathrm{G}}},\ \frac{n_{\mathrm{a}}+ i}{n_{\mathrm{A}}}\right),\ i = - m,\dots, - 1,\ 0,\ 1,\dots, n $$

where the constraints are *n*_g_ – *m* ≥ 0, *n*_a_ – *m* ≥ 0, *n*_g_ + *n* ≤ *n*_G_, *n*_a_ + *n* ≤ *n*_A_, and *n*_G_ – *n*_g_ = *n*_A_ – *n*_a_.

The averages of the FN rate and the FP rate are,

12 $$ \begin{array}{l}{\overline{r}}_{\mathrm{fn}} = \frac{1}{m+ n+1}\ {\displaystyle \sum_{i = - m}^n}\frac{n_{\mathrm{g}}+ i}{n_{\mathrm{G}}} = \frac{n_{\mathrm{g}}}{n_{\mathrm{G}}} + \frac{1}{n_{\mathrm{G}}} \times \frac{n- m}{2}\\ {}{\overline{r}}_{\mathrm{fp}} = \frac{1}{m+ n+1}\ {\displaystyle \sum_{i = - m}^n}\frac{n_{\mathrm{a}}+ i}{n_{\mathrm{A}}} = \frac{n_{\mathrm{a}}}{n_{\mathrm{A}}} + \frac{1}{n_{\mathrm{A}}} \times \frac{n- m}{2}\ .\end{array} $$

Hence, the correlation coefficient is,

13 $$ \begin{array}{l}\rho =\frac{{\displaystyle {\sum}_{i = - m}^n}\left(\ {r}_{\mathrm{f}\mathrm{n}\  i} - {\overline{r}}_{\mathrm{f}\mathrm{n}}\ \right)\ \left(\ {r}_{\mathrm{f}\mathrm{p}\  i} - {\overline{r}}_{\mathrm{f}\mathrm{p}}\ \right)}{\sqrt{{\displaystyle {\sum}_{i = - m}^n}{\left(\ {r}_{\mathrm{f}\mathrm{n}\  i} - {\overline{r}}_{\mathrm{f}\mathrm{n}}\ \right)}^2}\ \sqrt{{\displaystyle {\sum}_{i = - m}^n}{\left(\ {r}_{\mathrm{f} p\  i} - {\overline{r}}_{\mathrm{f} p}\ \right)}^2}}\\ {} = \frac{{\displaystyle {\sum}_{i = - m}^n}\left(\  i - \frac{n- m}{2}\ \right)\left(\  i - \frac{n- m}{2}\ \right)}{\sqrt{{\displaystyle {\sum}_{i = - m}^n}{\left(\  i - \frac{n- m}{2}\ \right)}^2}\ \sqrt{{\displaystyle {\sum}_{i = - m}^n}{\left(\  i - \frac{n- m}{2}\ \right)}^2}}=1\ .\end{array} $$

Further, using the first formula of Eq. (3), the SE of the average MER *r*_a_ turns out to be SÊ_a_ = (SÊ_fn_ + SÊ_fp_) / 2, in which SÊ_fn_ and SÊ_fp_ may be estimated using SÊ = sqrt [$\widehat{r}$ (1 – $\widehat{r}$) / *n*], where $\widehat{r}$ = *r*_fn_ and *n* = *n*_G_ for SÊ_fn_, and $\widehat{r}$ = *r*_fp_ and *n* = *n*_A_ for SÊ_fp_.

However, such an analytical approach generally underestimates the SE of MER, and thus the SE of TER (see Eq. (7)) as opposed to the bootstrap method. For the seven CIS algorithms, if the bootstrap method is employed, the estimated SÊs of TER, in which the average MER *r*_a_ is used, are 0.001001, 0.000246, 0.000537, 0.000254, 0.001888, 0.000820, and 0.000292, respectively. If the analytical approach is used, they are 0.000169, 0.000181, 0.000180, 0.000188, 0.000196, 0.000219, and 0.000208, respectively.

## Conclusions

Our novel performance measure TER comes with SE and 95% CI without restrictions on data size, and the correlation coefficient of TERs between two CIS algorithms is also solved. Thus, the well-established statistical approach can be carried out to evaluate and compare the performance levels of CIS algorithms with statistical confidence. Significance values for differences in CIS algorithm performance in combination with other factors such as computational execution time, etc. can be used as a basis for selecting algorithms.

No matter which MER is chosen and no matter which CIS algorithms and datasets are employed, the approaches of designing the TER using the total probability in statistics based on MER, computing SE and 95% CI of TER based on using the bootstrap method to estimate the SE of MER, and conducting hypothesis testing, etc. explored in this article remain intact. The TER *ε* aggregates all MERs weighted by the size of a cell divided by the total sizes of all cells so that the algorithm pays more penalties if making errors while segmenting larger cells.

The TER *ε* is supported by the pairwise-comparison test of MERs using 106 manually segmented GT cells with different sizes and seven CIS algorithms taken from ImageJ. It is also qualitatively consistent with the observations from the MER histograms. The TER approach is more effective than the bivariate approach and the CDF approach.

The SE and 95% CI of the TER are computed using Eq. (7), based on the SE of MER that is calculated using the bootstrap method under a constraint condition for CIS during bootstrap resampling. The nature of the bootstrap method is stochastic. However, our studies reveal that the variation of the estimated SÊ of TER is small. Moreover, in our studies, all estimated SÊs of TERs obtained by a random execution of bootstrap method while computing the SÊs of MERs fall in the 95% CÎ of the estimated SÊs of TERs correspondingly.

The error bars of the TERs can be used to evaluate the performance level of a CIS algorithm against a hypothesized value, and classify CIS algorithms into different classes in terms of performance accuracies based on the criteria of performance accuracies. While the error bars overlap, the two-algorithm hypothesis testing can be employed to compare two CIS algorithms and determine the statistical significance of their performance difference. The Z test in Eq. (9) involves not only the SEs of TERs but also the correlation coefficient between the TERs of two CIS algorithms, which are all investigated in this article.

## Abbreviations

AD: Algorithm detected
CDF: Cumulative distribution function
CI: Confidence interval
CIS: Cell image segmentation
FN: False negative
FP: False positive
GT: Ground truth
MER: Misclassification error rates
ROC: Receiver operating characteristic
SE: Standard error
TER: Total error rate
WR: With replacement

## Footnotes

1 Certain commercial entities, equipment, or materials may be identified in this document in order to describe an experimental procedure or concept adequately. Such identification is not intended to imply recommendation or endorsement by the National Institute of Standards and Technology, nor is it intended to imply that the entities, materials, or equipment are necessarily the best available for the purpose.
